# Elucidating the role of *Rhodiola rosea* L. in sepsis-induced acute lung injury via network pharmacology: emphasis on inflammatory response, oxidative stress, and the PI3K-AKT pathway

**DOI:** 10.1080/13880209.2024.2319117

**Published:** 2024-03-06

**Authors:** Lu Jiang, Dongdong Yang, Zhuoyi Zhang, Liying Xu, Qingyu Jiang, Yixin Tong, Lanzhi Zheng

**Affiliations:** a Department of Emergency, The First Affiliated Hospital of Zhejiang Chinese Medical University (Zhejiang Provincial Hospital of Chinese Medicine), Hangzhou, Zhejiang, China; b Department of Medical Administration, The First Affiliated Hospital of Zhejiang Chinese Medical University (Zhejiang Provincial Hospital of Chinese Medicine), Hangzhou, Zhejiang, China

**Keywords:** *Rhodiola rosea* L, sepsis-induced acute lung injury, pulmonary fibrosis, inflammation, network pharmacology, molecular docking, experimental verification, PI3K-AKT signalling pathway

## Abstract

**Context:**

Sepsis-induced acute lung injury (ALI) is associated with high morbidity and mortality. *Rhodiola rosea* L. (Crassulaceae) (RR) and its extracts have shown anti-inflammatory, antioxidant, immunomodulatory, and lung-protective effects.

**Objective:**

This study elucidates the molecular mechanisms of RR against sepsis-induced ALI.

**Materials and methods:**

The pivotal targets of RR against sepsis-induced ALI and underlying mechanisms were revealed by network pharmacology and molecular docking. Human umbilical vein endothelial cells (HUVECs) were stimulated by 1 μg/mL lipopolysaccharide for 0.5 h and treated with 6.3, 12.5, 25, 50, 100, and 200 μg/mL RR for 24 h. Then, the lipopolysaccharide-stimulated HUVECs were subjected to cell counting kit-8 (CCK-8), enzyme-linked immunosorbent, apoptosis, and Western blot analyses. C57BL/6 mice were divided into sham, model, low-dose (40 mg/kg), mid-dose (80 mg/kg), and high-dose (160 mg/kg) RR groups. The mouse model was constructed through caecal ligation and puncture, and histological, apoptosis, and Western blot analyses were performed for further validation.

**Results:**

We identified six hub targets (MPO, HRAS, PPARG, FGF2, JUN, and IL6), and the PI3K-AKT pathway was the core pathway. CCK-8 assays showed that RR promoted the viability of the lipopolysaccharide-stimulated HUVECs [median effective dose (ED_50_) = 18.98 μg/mL]. Furthermore, RR inhibited inflammation, oxidative stress, cell apoptosis, and PI3K-AKT activation in lipopolysaccharide-stimulated HUVECs and ALI mice, which was consistent with the network pharmacology results.

**Discussion and conclusion:**

This study provides foundational knowledge of the effective components, potential targets, and molecular mechanisms of RR against ALI, which could be critical for developing targeted therapeutic strategies for sepsis-induced ALI.

## Introduction

Sepsis, caused by an abnormal host response to infection, is a life-threatening and severe disease syndrome (Alsharif et al. 2021). It is estimated that over 19 million individuals worldwide are affected by sepsis each year, resulting in approximately 6 million deaths and a mortality rate exceeding 25% (Pei et al. 2022). Sepsis usually causes organ failure, with the lungs commonly being the first organ to be affected. More than half of sepsis patients develop either acute lung injury (ALI) or acute respiratory distress syndrome (Sevransky et al. 2009). The progression of sepsis-induced ALI involves the activation of inflammatory and apoptotic pathways, which results in alveolar epithelial cell destruction, increased epithelial permeability, and the accumulation of oedema fluid in the alveolar space (Qiu et al. 2020). ALI is characterized by oedema, hyperaemia, and infiltration of inflammatory cells, representing a severe form of lung injury (Yang R et al. 2021). Currently, the primary methods of treating sepsis and ALI are antibiotics and supportive measures; however, their effectiveness is still unsatisfactory (Jiang et al. 2021). Therefore, there is an urgent need to identify an effective and reliable drug to treat sepsis-induced ALI.

In light of the current therapeutic limitations, research is increasingly focusing on alternative treatments, such as medicinal plants. These plants and their derivatives offer a wealth of potential therapeutic agents. Indeed, the World Health Organization (WHO) reports that around 80% of the global population relies on plant-based components and their active constituents for traditional remedies (Shahat et al. 2018; Alqahtani et al. 2022). *Rhodiola rosea* L. (Crassulaceae) (RR), also known as ‘golden root’ or ‘roseroot’, is a traditional Chinese medicine (TCM) with various biological functions, such as antidiabetes, anticancer, anti-aging, antioxidation, anti-inflammation, and immune regulation (Tao et al. 2019; Pu et al. 2020). For example, salidroside, an active compound of RR, is a potent anti-diabetic agent that inhibits adipogenesis and inflammation in epididymal white adipose tissue and stimulates leptin signalling in the hypothalamus (Wang M et al. 2016). A prior study reported that *Rhodiola crenulata* extract contains components with oestrogenic activity, and prolonged treatment reduces the transcriptional activity of β-catenin and the oestrogen receptor response, leading to reduced proliferation and tumorsphere formation (Bassa et al. 2016). RR has also been found to exert anti-inflammatory effects by inhibiting the leukotriene signalling pathway (Panossian et al. 2019). In addition, the majority of studies have reported that RR has therapeutic effects on lung diseases, including pulmonary arterial hypertension (Ren et al. 2021), lung fibrotic injury (Zhang K et al. 2016), and hypoxic pulmonary oedema (Lee et al. 2013), and so on. More importantly, some studies have reported that RR and its extracts can ameliorate ALI (Xu et al. 2003; Song et al. 2021; Zhang H et al. 2021), particularly sepsis-induced ALI (Lan et al. 2017). A previous study found that RR could attenuate ALI and inhibits NF-ĸB activation in septic mice (Zhang Y et al. 2017). However, the mechanism by which RR treats sepsis-induced ALI remains largely unexplored.

Network pharmacology is an emerging field that aims to understand disease and drug mechanisms within the broader context of biological networks. This approach aligns closely with the core principles of TCM, which emphasize multi-target and pathway-oriented therapies (Zhang GB et al. 2013). In summary, our study aims to elucidate the molecular mechanisms by which RR treats sepsis-induced ALI, employing a combination of network pharmacology techniques, molecular docking, and empirical testing. These findings could pave the way for the development of more effective treatments for this severe medical condition.

## Materials and methods

### Collection of sepsis-induced ALI-related targets

Sepsis-induced ALI-related targets were obtained from DisGeNET (Piñero et al. 2017), GeneCards (Safran et al. 2010), and OMIM (Online Mendelian Inheritance in Man) (Amberger and Hamosh 2017) databases using the search terms ‘sepsis’ and ‘acute lung injury’.

### Screening for potential targets of active ingredients in RR

The active ingredients in RR were collected by reviewing the literature (Liu M et al. 2006; Yuan et al. 2007; Chai et al. 2015; Yang W et al. 2015; Wang J and Xu 2016; Zhang J et al. 2016) and then standardized to their respective names using the PubChem database. Compounds without 2D or 3D structures were excluded from the study.

The SwissADME (absorption, distribution, metabolism, and excretion) database (Daina et al. 2017) was used to analyse the drug-likeness (DL) of the compounds. The selected active compounds from RR met the following criteria: high gastrointestinal (GI) absorption, meeting at least two of the five DL assessment methods, and oral bioavailability (OB) ≥30%.

Active components in a drug produce biological outcomes by interacting with molecular targets. The target prediction of the active compounds was based on the Similarity ensemble approach (SEA) (Keiser et al. 2007), where the species included was ‘human,’ and the potential targets of active compounds were obtained with an adjusted *p*-value ≤0.01.

### Protein-protein interaction (PPI) network construction

To further identify the core targets, overlapping targets related to RR and sepsis-induced ALI were obtained using Venny 2.1. These targets were then placed into the STRING database, limited to *Homo sapiens.* Subsequently, the PPI results were imported into the Cytoscape 3.8.0 software (Shannon et al. 2003) to construct the PPI network. Then, a plug-in CytoHubba (Chin et al. 2014) in the Cytoscape software was used to select hub genes in the PPI network according to degrees.

### Enrichment analysis

To study the function and potential mechanisms of the core targets, Gene Ontology (GO) and Kyoto Encyclopedia of Genes and Genomes (KEGG) pathway enrichment analysis was conducted with the R packages ‘DOSE version 3.13.2’ and ‘clusterProfiler version 3.15.4’. A *p*-value < 0.05 was set as the criterion for significant differences.

### Active components analysis

The main active components of RR [purchased from Nordicon Weiguang Pharmaceutical Co., Ltd. (Sichuan, China)] were analysed using HPLC-MS. Eclipse Plus C18 RRHD (1.8 μm, 2.1 × 50 mm) (Agilent Technologies, DE, USA) was utilized for separating samples, with the temperature maintained at 30 °C and flow rate of 0.4 mL/min. The mobile phase was composed of 0.1% formic acid (55%) and methanol (45%). Each sample had an injection volume of 1 µL. Positive and negative ion modes were used for mass spectrometry. For the positive ion mode, the gas pressure was set to 40 psi, and the ion spray voltage was 500 V at a temperature of 350 °C. For the negative ion mode, the ion spray voltage was set at −1500 V, while the remaining parameters were consistent with those in the positive mode.

### Molecular docking

The top nine bioactive component chemical structures were obtained from the ZINC website (Sterling and Irwin 2015). These structures were charged, and rotatable bonds were assigned using AutoDock Tools 1.5.6. The top eight hub targets’ crystal structures were acquired from the RCSB Protein Data Bank (PDB) (Burley et al. 2017). Co-crystal ligands and active pockets for each target were identified using AutoDockTools 1.5.6. Molecular docking of the active compounds with their potential targets and the calculation of their free binding energies (affinity) were carried out using Autodock Vina 1.1.2 software (Trott and Olson 2010). The results were visualized and analysed using PyMOL 2.3.0 software (Seeliger and de Groot 2010).

### Cell culture and treatment

Human umbilical vein endothelial cells (HUVECs) were acquired from the Chinese Academy of Sciences Shanghai Cell Bank (CVCL number: B7UI; catalogue number: GNHu39; Shanghai, China) and cultured in Dulbecco’s modified Eagle’s medium (DMEM; Gibco, Life Technologies, USA) supplemented with 10% foetal bovine serum and 1% penicillin-streptomycin at 37 °C in a 5% CO_2_ environment. HUVECs were seeded into a culture dish for 12 h, treated with 1 μg/mL lipopolysaccharide (LPS; Sigma, CA, USA) for 0.5 h, and then treated with 6.3, 12.5, 25, 50, 100, and 200 μg/mL RR for 24 h.

### Cell viability assay

The Cell Counting Kit-8 (CCK-8) was used to determine cell viability. Briefly, HUVECs were seeded into 96-well plates (1 × 10^5^ cells/well). After LPS stimulation and subsequent RR (Nordicon Weiguang Pharmaceutical Co., Ltd., Sichuan, China) treatment for 24 h, 10 μL CCK-8 solution (Beyotime, China) was introduced to each well. The mixture was then incubated at 37 °C for 1 h. Absorbance was recorded on a DR-200Bs microplate reader (Diatek, China) at a wavelength of 450 nm.

**Figure 1.**
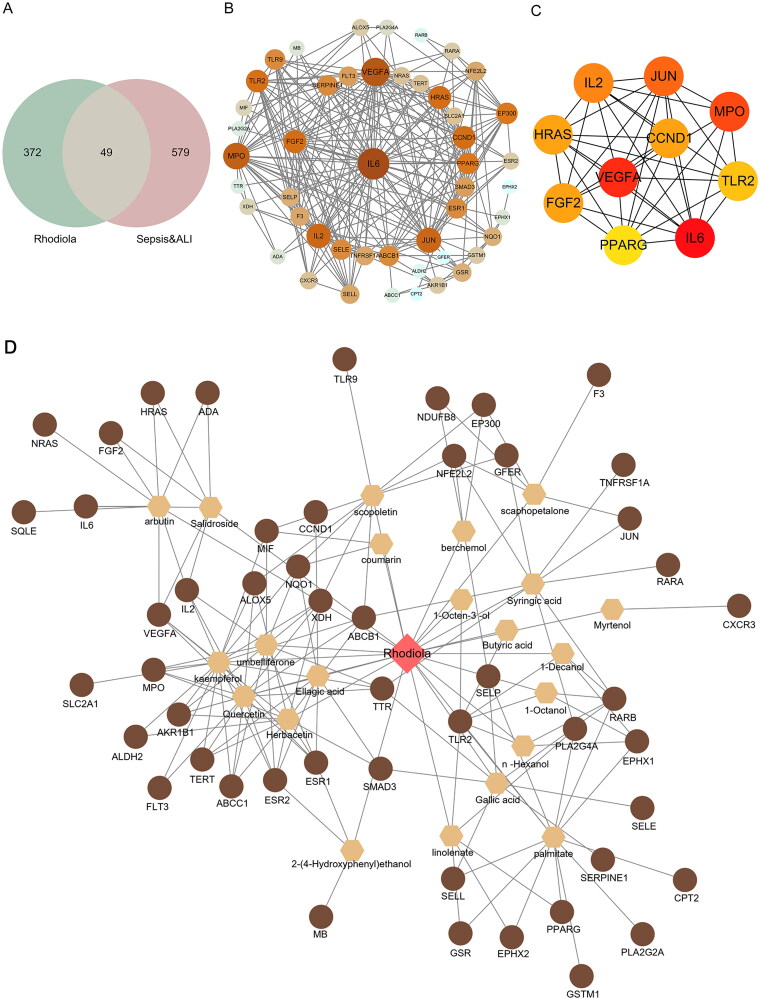
Drug-compounds-targets (D-C-T) network. (A) Venn diagram of active ingredients of RR and disease-related targets. (B) Protein-protein interaction (PPI) network diagram. The size of the node is proportional to the degree, and each edge represents the interaction between the compound molecule and the target. (C) The top 10 hub genes network. (D) D-C-T network diagram. The brown circles in the diagram are the key targets of RR acting on the disease, the hexagons represent active compounds and the red quadrilateral represents RR. The size of a node is proportional to its degree value.

### Animals and model establishment

Thirty C57BL/6 mice (eight weeks old) were purchased from the Comparative Medicine Research Institute of Yangzhou University. To construct an *in vivo* model of sepsis-induced ALI, mice underwent caecal ligation and puncture surgery as per previous research (Chen R et al. 2022). Briefly, mice in the ALI group were anesthetized using intraperitoneal injection of pentobarbital sodium (50 mg/kg) (Wu et al. 2018) and an incision of approximately 2 cm was made along the abdominal midline to expose the caecum. Approximately one-third of the caecum was tied off using a 5-0 suture, followed by two punctures with a 21-gauge sterilized needle. A small amount of intestinal content was expelled from the puncture hole by gentle squeezing to ensure patency. The caecum was relocated, and the wound was sutured. Mice in the sham group underwent similar surgery; however, their caecum was not ligated or punctured. All mice received a subcutaneous injection of 5 mL/100 g saline for fluid resuscitation post-surgery.

The caecal ligation and puncture-induced mice were randomly divided into four groups (*n* = 6) (Chen R et al. 2022): model group, low-dose RR group, mid-dose RR group, and high-dose RR group. RR-treated mice received oral gavage of RR solution at doses of 40 (low-dose), 80 (mid-dose), and 160 (high-dose) mg/kg daily for seven days, whereas the sham and model groups received equivalent volumes of saline. Five days after the last RR treatment, all mice were euthanized by CO_2_ inhalation following Zoletil anaesthesia, and efforts were made to minimize animal distress. The death was validated by confirming cardiac and respiratory arrest. The animal euthanasia was executed by Zhuoyi Zhang. Tissue and serum samples were collected for follow-up testing. All animal experiments were approved by the Experimental Animal Ethics Committee of Yangzhou University (202303130), which conformed to the Guide for the Care and Use of Laboratory Animals. The animal experiments were conducted by Dongdong Yang from 25 April 2023 to 29 May 2023.

### Histological analysis

Haematoxylin-eosin (HE) staining was performed to assess lung injury. Lung tissue samples were fixed in 4% paraformaldehyde, hydrated through a graded series of ethanol concentrations, embedded in paraffin, and sectioned into 4-μm thick slices. The slices were deparaffinized in xylene solution and stained with haematoxylin and eosin. Images were captured under a microscope (Olympus, Tokyo, Japan).

### Apoptosis analysis

Apoptosis in HUVECs was identified using flow cytometry. The apoptotic cells were isolated from the living cells using an annexin V-fluorescein isothiocyanate/propidium iodide dual staining kit and examined with a FACScan Flow Cytometer (Becton-Dickinson, NJ, USA).

A TUNEL kit (Abcam, UK) was used to detect the levels of apoptosis in the lung tissue. Briefly, lung tissue was sectioned and incubated with 50 μL of TUNEL solution for 60 min at 37 °C in the dark and then with DAPI for 10 min in the dark. Five areas were randomly selected for microscopic observation (Olympus, Japan).

### Enzyme-linked immunosorbent assay (ELISA)

The levels of reactive oxygen species (ROS), malondialdehyde (MDA), superoxide dismutase (SOD), myeloperoxidase (MPO), tumour necrosis factor-α (TNF-α), interleukin-6 (IL-6), and interleukin-1β (IL-1β) were determined using ELISA kits (Thermo Fisher Scientific, MD, USA) following the manufacturer’s instructions. The ELISA kits were equilibrated at room ­temperature for 30 min, and then the concentrated washing solution was diluted 20 times with distilled water. Subsequently, the prepared enzyme-labelling working solution was then added and incubated at 37 °C in a water bath for another 30 min. Colour development was achieved by adding colour reagent A and B, mixing thoroughly, and incubating at 37 °C in the dark for 15 min. Finally, optical density at 450 nm was assessed using a microplate reader (DR-3518G, Hiwell Diatek, Wuxi, China). The following ELISA kits were employed: ROS (human; catalogue number: A098751, Affandi-e, Shanghai, China), MDA (human; catalogue number: EH4174, Fine Test, Wuhan, China), SOD (human; catalogue number: MM-0390H1, Jiangsu Meimian, China), MPO (human; catalogue number: MM-2467H1, Jiangsu Meimian), TNF-α (human; catalogue number: ab181421, Abcam), IL-6 (human; catalogue number: MM-0049H1, Jiangsu Meimian), IL-1β (human; catalogue number: ab214025, Abcam); MDA (mouse; catalogue number: EM1723-1, Fine Test), ROS (mouse; catalogue number: SP14834, Spbio, Wuhan, China), SOD (mouse; catalogue number: MM-0389M1, Jiangsu Meimian), MPO (mouse; catalogue number: MM-0338M1, Jiangsu Meimian), TNF-α (mouse; catalogue number: ab208348, Abcam), IL-6 (mouse; catalogue number: ab216165, Abcam), IL-1β (mouse; catalogue number: MM-0163M1, Jiangsu Meimian).

### Western blot test

Total protein was extracted using radioimmuno-precipitation assay (RIPA) lysis buffer (Beyotime, Shanghai, China). Then, a bicinchoninic acid (BCA) kit was used to quantify protein concentration and separated by gel electrophoresis (Beyotime). The loading volume was 20 μg. Following this, protein samples were transferred onto membranes. Membranes were sealed with bovine serum albumin (BSA) (Beyotime) for 1 h and incubated with primary antibodies at 4 °C overnight, followed by incubation with secondary antibody (1:2000, ab9485, Abcam) for 1 h. Protein bands were subsequently detected using enhanced chemiluminescent reagents (Millipore). Protein expression was quantified using ImageJ software, with GAPDH serving as the loading control. The primary antibodies used were as follows: anti-PI3K (human and mouse; dilution: 1:1000; catalogue number: ab151549, Abcam, UK), anti-p-PI3K (human and mouse; dilution: 1:1000; catalogue number: ab182651, Abcam), anti-AKT (human and mouse; dilution: 1:500; catalogue number: ab8805, Abcam), anti-p-AKT (human and mouse; dilution: 1:500; catalogue number: ab38449, Abcam), and anti-GAPDH (human and mouse; dilution: 1:3000; catalogue number: AF7021, Affinity, USA).

### Statistical analyses

Resulting data are shown as mean ± standard deviation. Statistical analyses were conducted using SPSS software (version 25.0; IBM, NY, USA). One-way ANOVA followed by Tukey’s test was employed for multiple group comparisons. A *p*-value of < 0.05 was considered to indicate statistical significance.

## Results

### Sepsis-induced ALI-related targets

Sepsis-induced ALI-related targets were retrieved from public databases, resulting in 152 sepsis-related targets and 93 ALI-related targets from the DisGeNET database, 291 sepsis-related targets and 313 ALI-related targets from the GeneCards database, and 2 sepsis-related targets and 26 ALI-related targets from the OMIM database. A total of 628 disease-target genes were identified after removing duplicate targets.

### Active ingredients and potential targets of RR

After searching the literature, we collected 49 active ingredients of RR. Thirty ingredients were retained after the DL screening (OB ≥30%). A total of 421 potential targets of the active compounds were predicted using SEA, restricted to the human species, and adjusted for *p*-values ≤0.01.

### PPI and hub genes network construction

We input 628 sepsis-induced ALI-related targets and 421 active compound-related targets into Venny 2.1 and obtained 49 overlapping targets (Figure 1(A) and Table S1). Subsequently, we constructed a PPI network for 49 overlapping targets using the STRING database and Cytoscape 3.8.0 software. This network contained 48 nodes (excluding one non-interacting target) and 262 edges (Figure 1(B)).

Furthermore, the top 10 targets with high degrees were selected as hub genes, including VEGFA, CCND1, MPO, TLR2, IL6, PPARG, FGF2, HRAS, IL2, and JUN. A PPI network of hub genes containing 10 nodes and 39 edges was constructed using the Cytoscape 3.8.0 software (Figure 1(C)).

### Drug-compounds-targets (D-C-T) network construction

Based on the 49 overlapping targets, we further determined that RR action against sepsis-induced ALI was primarily associated with 22 main active components. Information on the 22 main active components is presented in Table S2.

In addition, a D-C-T network was obtained by importing the 22 main active components and 49 key targets of RR into Cytoscape 3.8.0 software, as shown in Figure 1(D). There were 73 nodes (RR, 22 main active components, and 49 key targets) and 163 edges, and the size of a node was proportional to its degree value. The brown circles in the network represent the 49 key targets of RR acting on the disease, the hexagons represent active compounds, and the red quadrilateral represents RR. These active compounds and their targets may play important roles in the action of RR in sepsis-induced ALI.

### GO and KEGG enrichment analyses

**Figure 2.**
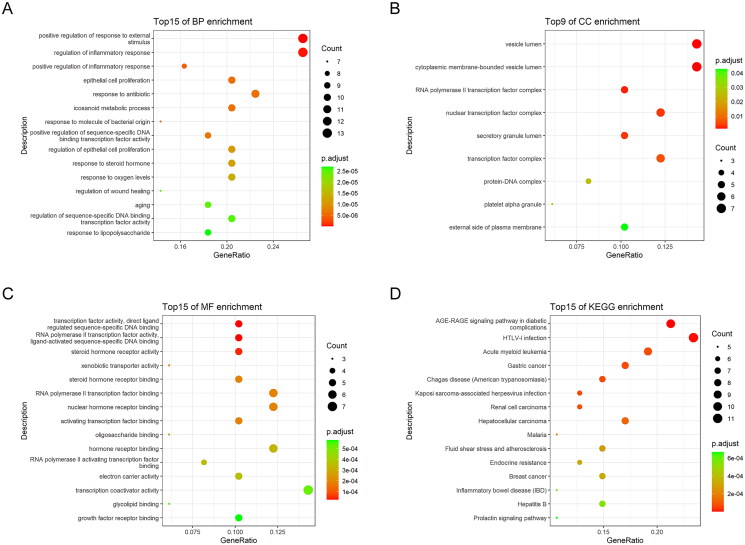
GO and KEGG enrichment analyses of 49 key targets. GO enrichment analysis of key targets in biological process (BP) (a), cellular component (CC) (B), and molecular function (MF) (C). KEGG pathway enrichment analysis of key targets (D).

To obtain a more detailed understanding of the potential mechanisms of RR acting on sepsis-induced ALI, GO and KEGG enrichment analyses were performed on the 49 key targets. GO annotation showed that the 49 key targets were classified into 751 biological processes (BP) terms, mainly related to positive regulation of response to external stimulus and regulation of inflammatory response (Figure 2(A)); 9 cellular components (CCs) terms, mainly located in the vesicle lumen and cytoplasmic membrane-bounded vesicle lumen (Figure 2(B)); and 64 molecular function (MF) terms, mainly related to transcription factor activity, direct ligand-regulated sequence-specific DNA binding and RNA polymerase II transcription factor activity, ligand-activated sequence-specific DNA binding (Figure 2(C)) (*p*** **<** **0.05).

**Figure 3.**
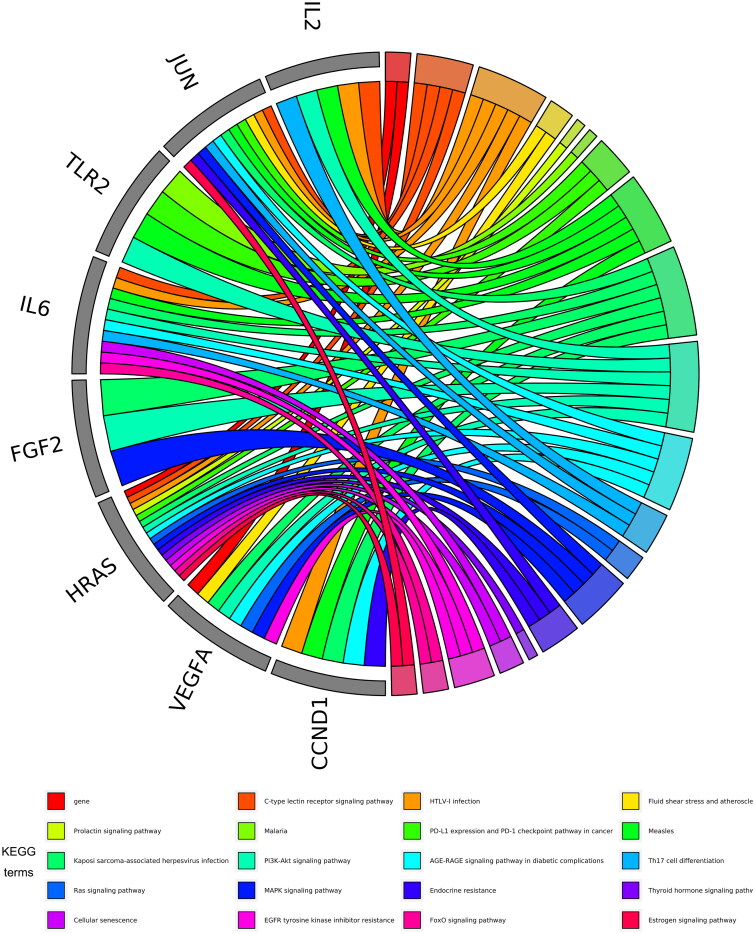
The top 20 pathways of hub genes.

**Figure 4.**
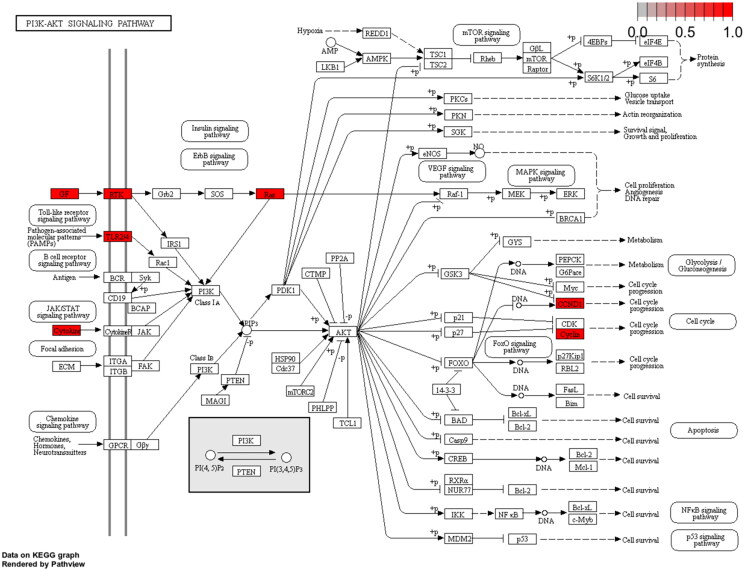
Map of the PI3K-AKT signalling pathway.

KEGG pathway analysis showed that 49 key targets were classified into 83 KEGG pathways (*p*** **<** **0.05). As shown in Figure 2(D), the targets were predominantly associated with HTLV-1 infection, the AGE-RAGE signalling pathway in diabetic complications, and Kaposi sarcoma-associated herpesvirus infection. In addition, the hub genes participating in the greatest number of pathways were JUN, IL6, and HRAS (Figure 3). Furthermore, PI3K-AKT had the highest number of targets in addition to the three pathways mentioned above. Therefore, we mapped the PI3K-AKT signalling pathway diagram with the key targets in the pathway represented by the red box (Figure 4).

### Molecular docking verification

To uncover the primary constituents of RR, we performed an analysis on RR utilizing HPLC-MS. We identified three major compounds within RR: salidroside, gallic acid, and kaempferol, with concentrations of 132 ng/mL, 258 ng/mL, and 195 ng/mL, respectively (Table 1 and Figure S1).

**Table 1.** The main ingredients of *Rhodiola rosea* L.

Analyte	Molecular formula	2D Structure	Ionization mode	Retention time (min)	Content (ng/mL)
Salidroside	C_14_H_20_O_7_	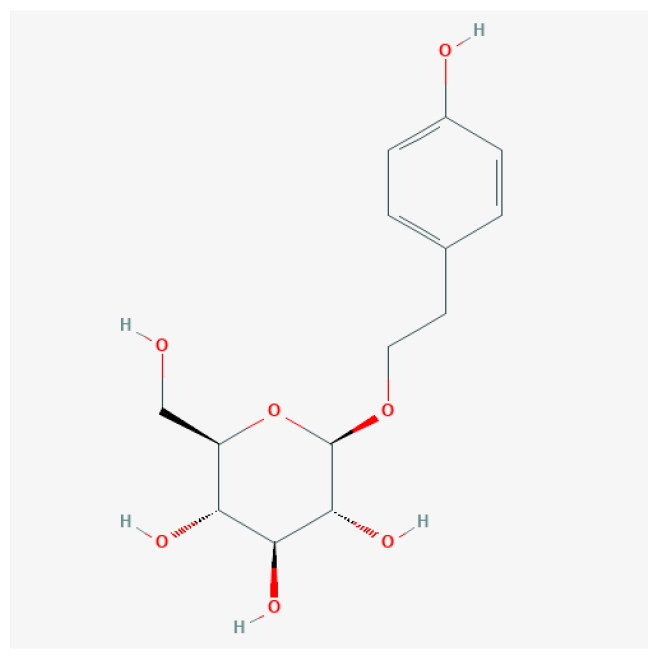	M + H	0.562	132
Gallic acid	C_7_H_6_O_5_	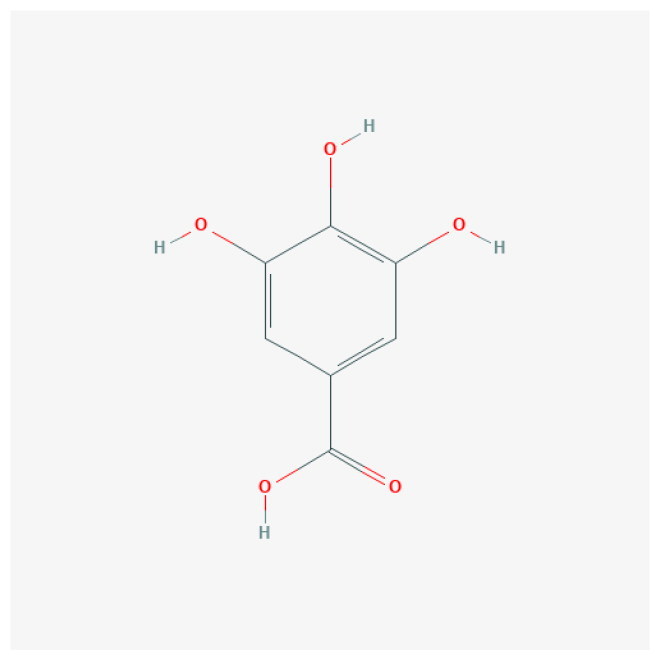	M-H	0.519	258
Kaempferol	C_15_H_10_O_6_	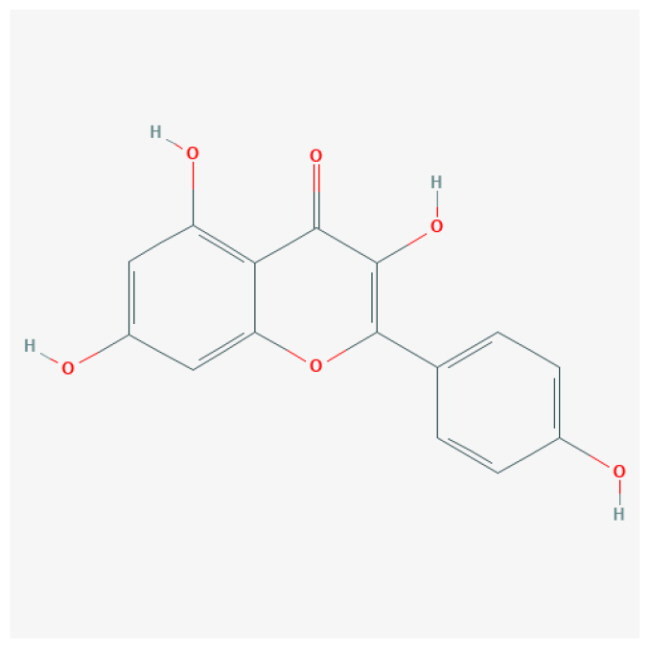	M-H	5.439	195

The top 8 hub targets (IL6, HRAS, MPO, CCND1, FGF2, TLR2, PPARG, and JUN) were chosen for molecular docking analysis with these three main compounds. In this investigation, an affinity threshold of less than −5 kcal/mol was established, yielding a total of 18 docking pairs (Table 2). All three compounds demonstrated high binding affinity with MPO, HRAS, PPARG, FGF2, JUN, and IL6. The binding sites between the compounds and target proteins are depicted in Figure S2.

**Table 2.** The results of molecular docking.

Compound	PDB	GENE	Best affinity (kcal/mol)
Salidroside	5mfa	MPO	−8.3
	6q21	HRAS	−8.2
	3e00	PPARG	−7.6
	1iil	FGF2	−7.5
	1s9k	JUN	−7
	1alu	IL6	−5.8
Gallic acid	5mfa	MPO	−6.7
	6q21	HRAS	−6.5
	3e00	PPARG	−5.0
	1iil	FGF2	−5.4
	1s9k	JUN	−5.4
	1alu	IL6	−5.9
Kaempferol	5mfa	MPO	−9.4
	6q21	HRAS	−8.5
	3e00	PPARG	−8
	1iil	FGF2	−7.8
	1s9k	JUN	−7
	1alu	IL6	−6.5

### Effects of RR on viability, apoptosis, oxidative stress, and inflammatory response of LPS-stimulated HUVECs

**Figure 5.**
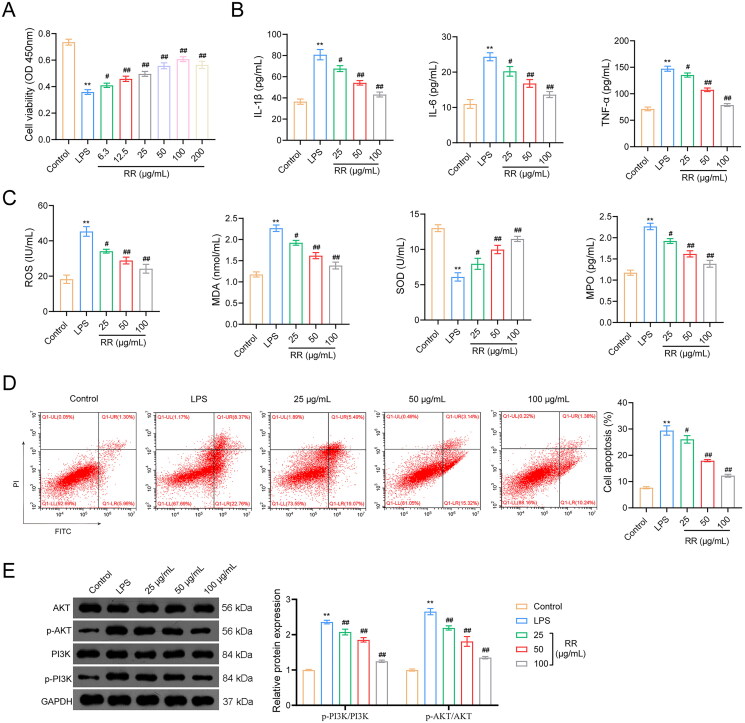
Effects of RR on the viability, apoptosis, oxidative stress, and inflammatory response in LPS-stimulated human umbilical vein endothelial cells (HUVECs). (A) the viability of HUVECs was detected by CCK-8. The contents of inflammatory factors (B) and oxidative stress factors (C) in LPS-stimulated HUVECs were performed by enzyme-linked immunosorbent assay (ELISA). (D) The apoptosis of HUVECs was detected by flow cytometry. (E) The expression of p-PI3K/PI3K and p-AKT/AKT in LPS-stimulated HUVEC cells were examined using Western blotting. **p < 0.01 vs. Control group; ^#^*p* < 0.05 and ^##^*p* < 0.01 vs. LPS group.

To verify the therapeutic effect of RR on sepsis-induced ALI, we constructed an *in vitro* ALI model using LPS-stimulated HUVECs. As shown in Figure 5(A), the viability of HUVECs significantly declined after LPS stimulation compared to the controls (*p*** **<** **0.01). RR treatment (ranging from 6.3 to 100 μg/mL) significantly increased the viability of LPS-stimulated HUVECs [*p*** **<** **0.05; median effective dose (ED50) = 18.98 μg/mL]. However, LPS-stimulated HUVECs viability decreased when RR concentration reached 200 μg/mL. Therefore, RR concentrations of 25, 50, and 100 μg/mL were chosen for subsequent experiments.

Moreover, network pharmacology indicated that oxidative stress and inflammation are associated with sepsis-induced ALI; therefore, we detected the expression of oxidative stress factors (MPO, ROS, MDA, and SOD) and inflammatory factors (TNF-α, IL-6, and IL-1β) in LPS-stimulated HUVECs. The expression of TNF-α, IL-6, and IL-1β significantly increased in the LPS group compared with that in the control group (*p*** **<** **0.01), and this facilitation was reversed by RR addition (*p*** **<** **0.05) (Figure 5(B)). In addition, LPS stimulation increased MPO, MDA, and ROS expression and decreased SOD expression (*p*** **<** **0.01). RR treatment dramatically downregulated MPO, MDA, and ROS expression and upregulated SOD expression in LPS-stimulated HUVECs (*p*** **<** **0.05) (Figure 5(C)).

To further explore the protective effects of RR on LPS-stimulated HUVECs, we used flow cytometry to detect apoptosis. As shown in Figure 5(D), LPS treatment increased the apoptosis of HUVECs, which was reversed by RR addition (*p*** **<** **0.01).

### RR inhibited activation of the PI3K-AKT pathway

The PI3K-AKT pathway is the classical inflammatory response pathway. In this study, we detected the expression of PI3K, p-PI3K, AKT, and p-AKT by western blotting. The results showed that LPS increased p-PI3K and p-AKT expression in HUVECs compared to that in control cells (*p*** **<** **0.01), whereas the stimulatory effects of LPS were reversed by RR intervention (*p*** **<** **0.01). The expression of PI3K and AKT did not change in any of the groups (Figure 5(E)).

### RR protected lung function and reduced sepsis-induced ALI

**Figure 6.**
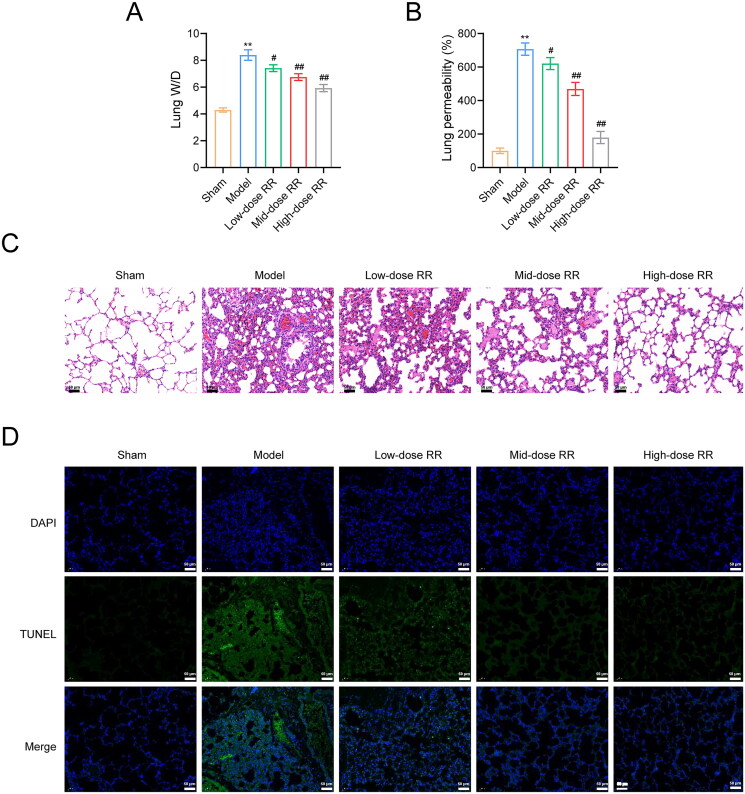
RR protected lung function and reduced sepsis-induced ALI *in vivo*. (A) The lung wet/dry (W/D) ratio. (B) Evans blue dye method was used to observe capillary permeability. (C) Haematoxylin-eosin (HE) staining for lung tissue pathological damage inspection. Scale bar: 50 μm. (D) The cell apoptosis in lung tissues using the TUNEL assay. Scale bar: 50 μm. **p < 0.01 vs. Sham group; #*p* < 0.05 and ##*p* < 0.01 vs. Model group.

**Figure 7.**
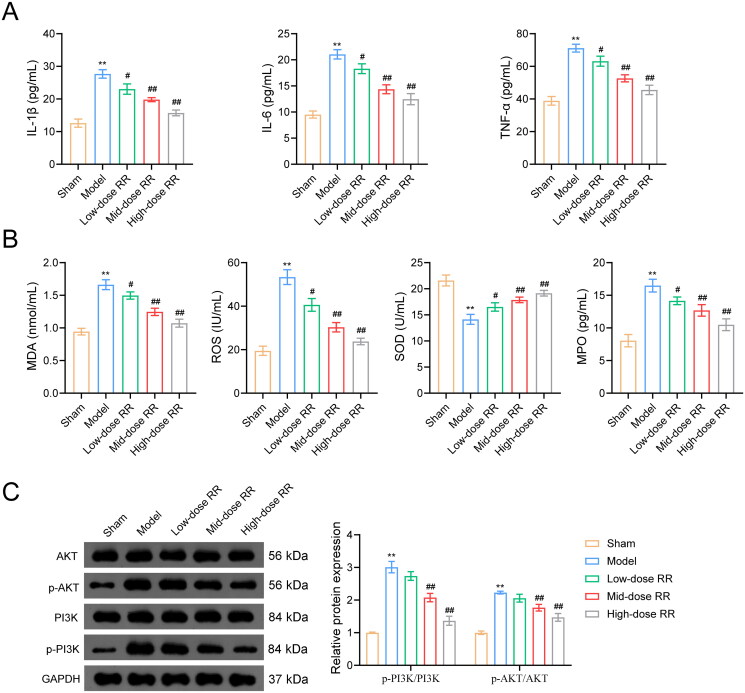
RR alleviated inflammation and oxidative stress, and inhibited activation of the PI3K-AKT pathway. ELISA assay was used to examine the effects of RR on the expression of inflammatory factors (A) and oxidative stress factors (B) in sepsis-induced ALI mice. (C) Western blot was used to detect the expression of p-PI3K/PI3K and p-AKT/AKT in lung tissues from sepsis-induced ALI mice. **p < 0.01 vs. Sham group; ^#^*p* < 0.05 and ^##^*p* < 0.01 vs. Model group.

To further verify the ability of RR to prevent sepsis-induced ALI, we constructed an ALI mouse model by caecal ligation and puncture. As shown in Figure 6(A), the lung wet/dry ratio decreased after RR treatment. To measure capillary function, we observed the permeability of lung tissues using the Evans blue dye method and found that the high permeability in the model group was reversed by RR (Figure 6(B)). Similarly, HE staining showed that lung tissues from ALI model mice exhibited obvious alveolitis, cellular infiltration in the alveolar cavity and pulmonary stroma, destroyed alveolar structures, thickened alveolar septa, and fibrosis formation. Compared to the model group, alveolitis and pulmonary fibrosis were significantly reduced in lung tissues from the RR-administered group, with less inflammatory cell infiltration and reduced alveolar septal thickness (Figure 6(C)). Furthermore, we examined apoptosis in mouse lung tissues using the TUNEL assay. Compared to the sham group, apoptosis was significantly increased in the model group, which was weakened by RR administration (Figure 6(D)). In addition, we examined the effect of RR on the expression of oxidative stress factors, inflammatory factors, and the PI3K-AKT signalling pathway in ALI mice *in vivo*. The ELISA results revealed that RR treatment reduced oxidative stress (decreased MPO, MDA, and ROS expression, increased SOD expression) and reduced the inflammatory response (decreased TNF-α, IL-6, and IL-1β expression) in ALI mice (*n* = 6; *p*** **<** **0.05) (Figure 7(A,B)). Western blotting showed that p-PI3K and p-AKT expression was significantly increased in the model group compared to the sham group, and RR addition reversed these phenomena (*n* = 6; *p*** **<** **0.05) (Figure 7(C)).

## Discussion

Sepsis is a severe clinical syndrome characterized by high morbidity and mortality. It is commonly linked with multiorgan failure and a dysregulated systemic inflammatory response (Iskander et al. 2013; Caraballo and Jaimes 2019). ALI is a notable complication of sepsis (Liu C et al. 2021). At present, there are no dedicated therapeutic drugs for sepsis-induced ALI. TCM has a long history of clinical practice and offers a diverse array of classical herbal formulas, serving as a valuable reservoir for drug discovery (Chao et al. 2017). RR, a kind of TCM, has been used for millennia. Although previous studies have shown that RR ameliorates sepsis-induced ALI (Lan et al. 2017), the underlying mechanisms remain poorly understood.

In this investigation, the role of RR in treating sepsis-induced ALI was rigorously examined. A total of 22 active ingredients and 49 pivotal targets by consulting public databases and relevant scientific literature. To elucidate the interactions among these targets, a PPI network was constructed, highlighting key proteins such as VEGFA, CCND1, MPO, TLR2, IL6, PPARG, FGF2, HRAS, IL2, and JUN. Further, GO enrichment analysis indicated that these 49 key targets modulate the inflammatory response. Additionally, KEGG pathway analysis indicated that these key targets are involved in 83 distinct pathways, including but not limited to the AGE-RAGE signalling pathway in diabetic complications and the PI3K-Akt signalling pathway. HPLC-MS was employed to further delineate the molecular constituents of RR, revealing three major compounds: salidroside, gallic acid, and kaempferol. Molecular docking studies demonstrated stable binding affinity of these compounds to six key proteins: MPO, HRAS, PPARG, FGF2, JUN, and IL6. Conclusively, the efficacy of RR in ameliorating sepsis-induced ALI was verified through both *in vitro* and *in vivo* experiments. These effects were associated with the modulation of the inflammatory response, the reduction of oxidative stress, and the regulation of the PI3K-AKT pathway. Collectively, this research lays a solid foundation for further investigations into the therapeutic potential and mechanisms of RR in the context of sepsis-induced ALI.

Sepsis-induced ALI is usually marked by a strong innate inflammatory reaction, leading to an overproduction of various inflammatory cytokines such as TNF-α, IL-6, and IL-1β (Chen Q et al. 2019). It has been demonstrated that sustainably increased concentrations of these inflammatory factors in plasma can increase mortality in patients with ALI (Qiu et al. 2020). Therefore, suppressing inflammation is an essential aspect of effective treatment for sepsis-induced ALI. Numerous studies have shown that RR and its active ingredients are effective in regulating the production of inflammatory mediators (Wei 2010; Guan et al. 2011; Huang and Hu 2017). Specific compounds extracted from RR—such as salidroside, kaempferol, gallic acid, and catechin—have been shown to inhibit the expression of multiple pro-inflammatory mediators, and thereby confer protective effects in murine models of sepsis and septic shock (Wei 2010). Salidroside has also been shown to attenuate inflammatory damage by decreasing the production of pro-inflammatory factors (such as TNF-α, IL-1β, and IL-6) (Guan et al. 2011). In our study, RR inhibited the production of pro-inflammatory factors both *in vivo* and *in vitro*. These findings underscore that the therapeutic efficacy of RR against sepsis-induced ALI is intrinsically linked to its anti-inflammatory properties.

Excessive oxidative stress plays an important role in the pathogenesis of ALI. Excess ROS production disrupts endothelial function and promotes pulmonary inflammation, further exacerbating ROS production—a destructive feedback loop that fuels ALI progression (Karki and Birukov 2019). A previous study showed that salidroside attenuates carbon tetrachloride-induced oxidative stress and exerts hepatoprotective effects through antioxidant activity by restoring hepatic glutathione, SOD, catalase, and MDA (Zhang X et al. 2020). Our investigation yielded a dose-dependent attenuation of oxidative stress markers such as MPO, MDA, and ROS, along with an elevation in SOD levels, in both LPS-stimulated HUVECs and ALI mouse models. These findings imply that the therapeutic potential of RR against sepsis-induced ALI may hinge on its antioxidant capabilities.

Evidently, RR treatment for ALI is associated with the restoration of excessive inflammatory response and oxidative stress. This corroborates our BP results, which indicate that the key targets of RR in GO analysis are chiefly implicated in inflammatory response and oxidative stress. In addition, KEGG analysis results indicated that RR treatment for ALI intersects with 83 pathways, notably encompassing the PI3K-AKT pathway. PI3K, a key member of the PI3K-AKT pathway, can be activated by multiple pathways to induce the secretion of pro-inflammatory cytokines and facilitate the recruitment of inflammatory cells to the lung (Ding et al. 2020). A previous study has reported that arctiin can prevent LPS-induced ALI in mice by inhibiting the PI3K/Akt pathway (Zhou et al. 2018). In our study, western blotting showed that RR treatment downregulated the phosphorylation levels of PI3K and AKT in LPS-stimulated HUVECs and ALI mice, suggesting that the anti-inflammatory action of RR in sepsis-induced ALI may operate *via* modulation of the PI3K-AKT pathway.

However, our study also has shortcomings. The intricate web of mechanisms through which RR acts on sepsis-induced ALI is expansive, and our investigation could only validate a subset thereof. For instance, other signalling pathways potentially influenced by RR remain unexplored. Secondly, while our study delves into certain bioactive components of RR, it remains conceivable that other, yet-unidentified active constituents may also contribute to its therapeutic efficacy. Finally, despite the general tolerability of RR, more extensive safety evaluations are warranted. Subsequent research should focus on its safety profile across different patient demographics, evaluate potential drug interactions, and identify any contraindications, particularly in the multifaceted clinical setting of sepsis-induced ALI.

## Conclusions

We identified 22 main components and 49 potential targets of RR for sepsis-induced ALI treatment using network pharmacology techniques. Moreover, molecular docking analysis further demonstrated that RR components have a good binding ability to hub targets. Furthermore, *in vitro* and *in vivo* experiments suggested that RR could reduce the pathological damage of sepsis-induced ALI and inhibit the production of pro-inflammatory factors and oxidative stress, probably by restraining the activation of the PI3K-AKT pathway. Our study provides a promising new drug for sepsis-induced ALI and a comprehensive reference for the mechanistic study of RR in sepsis-induced ALI.

## Supplementary Material

Supplemental Material

## Data Availability

The data that support the findings of this study are available from the corresponding author, [LZ], upon reasonable request.

## References

[CIT0001] Alqahtani AS, Ullah R, Shahat AA. 2022. Bioactive constituents and toxicological evaluation of selected antidiabetic medicinal plants of Saudi Arabia. Evid Based Complement Alternat Med. 2022:7123521–7123523. doi: 10.1155/2022/7123521. 35082904 PMC8786507

[CIT0002] Alsharif KF, Almalki AA, Alsanie WF, Alzahrani KJ, Kabrah SM, Elshopakey GE, Alghamdi AAA, Lokman MS, Sberi HA, Bauomy AA, et al. 2021. Protocatechuic acid attenuates lipopolysaccharide-induced septic lung injury in mice: the possible role through suppressing oxidative stress, inflammation and apoptosis. J Food Biochem. 45(10):e13915. doi: 10.1111/jfbc.13915. 34472624

[CIT0003] Amberger JS, Hamosh A. 2017. Searching online mendelian inheritance in man (OMIM): a knowledgebase of human genes and genetic phenotypes. Curr Protoc Bioinformatics. 58:1 2 1–1 2 12. 10.1002/cpbi.27 PMC5662200 28654725

[CIT0004] Bassa LM, Jacobs C, Gregory K, Henchey E, Ser-Dolansky J, Schneider SS. 2016. *Rhodiola crenulata* induces an early estrogenic response and reduces proliferation and tumorsphere formation over time in MCF7 breast cancer cells. Phytomedicine. 23(1):87–94. doi: 10.1016/j.phymed.2015.11.014. 26850689

[CIT0005] Burley SK, Berman HM, Kleywegt GJ, Markley JL, Nakamura H, Velankar S. 2017. Protein Data Bank (PDB): the single global macromolecular structure archive. Meth Mol Biol. 1607:627–641. 10.1007/978-1-4939-7000-1_26 PMC5823500 28573592

[CIT0006] Caraballo C, Jaimes F. 2019. Organ dysfunction in sepsis: an ominous trajectory from infection to death. Yale J Biol Med. 92(4):629–640. 31866778 PMC6913810

[CIT0007] Chai Y, Zhao G, Wang R, Wang M, Wu H, Tang S, Duan Q. 2015. Anti-tumor metastatic constituents from *Rhodiola wallichiana*. China J Chin Mater Med. 40:258–263. 26080555

[CIT0008] Chao J, Dai Y, Verpoorte R, Lam W, Cheng YC, Pao LH, Zhang W, Chen S. 2017. Major achievements of evidence-based traditional Chinese medicine in treating major diseases. Biochem Pharmacol. 139:94–104. doi: 10.1016/j.bcp.2017.06.123. 28636884

[CIT0009] Chen Q, Liu J, Wang W, Liu S, Yang X, Chen M, Cheng L, Lu J, Guo T, Huang F. 2019. Sini decoction ameliorates sepsis-induced acute lung injury via regulating ACE2-Ang (1-7)-Mas axis and inhibiting the MAPK signaling pathway. Biomed Pharmacother. 115:108971. doi: 10.1016/j.biopha.2019.108971. 31102910

[CIT0010] Chen R, Cao C, Liu H, Jiang W, Pan R, He H, Ding K, Meng Q. 2022. Macrophage Sprouty4 deficiency diminishes sepsis-induced acute lung injury in mice. Redox Biol. 62:102667. doi: 10.1016/j.redox.2023.102667. PMC9637958 36334381

[CIT0011] Chin CH, Chen SH, Wu HH, Ho CW, Ko MT, Lin CY. 2014. cytoHubba: identifying hub objects and sub-networks from complex interactome. BMC Syst Biol. 8(Suppl 4):S11. doi: 10.1186/1752-0509-8-S4-S11. 25521941 PMC4290687

[CIT0012] Daina A, Michielin O, Zoete V. 2017. SwissADME: a free web tool to evaluate pharmacokinetics, drug-likeness and medicinal chemistry friendliness of small molecules. Sci Rep. 7(1):42717. doi: 10.1038/srep42717. 28256516 PMC5335600

[CIT0013] Ding Q, Zhu W, Diao Y, Xu G, Wang L, Qu S, Shi Y. 2020. Elucidation of the mechanism of action of ginseng against acute lung injury/acute respiratory distress syndrome by a network pharmacology-based strategy. Front Pharmacol. 11:611794. doi: 10.3389/fphar.2020.611794. 33746744 PMC7970560

[CIT0014] Guan S, Feng H, Song B, Guo W, Xiong Y, Huang G, Zhong W, Huo M, Chen N, Lu J, et al. 2011. Salidroside attenuates LPS-induced pro-inflammatory cytokine responses and improves survival in murine endotoxemia. Int Immunopharmacol. 11(12):2194–2199. doi: 10.1016/j.intimp.2011.09.018. 22019446

[CIT0015] Huang Q, Hu XL. 2017. [Effects of salidroside on the secretion of inflammatory mediators induced by lipopolysaccharide in murine macrophage cell line J774.1.]. Sheng Li Xue Bao. 69:41–46. 28217806

[CIT0016] Iskander KN, Osuchowski MF, Stearns-Kurosawa DJ, Kurosawa S, Stepien D, Valentine C, Remick DG. 2013. Sepsis: multiple abnormalities, heterogeneous responses, and evolving understanding. Physiol Rev. 93(3):1247–1288. doi: 10.1152/physrev.00037.2012. 23899564 PMC3962548

[CIT0017] Jiang L, Ni J, Shen G, Xia Z, Zhang L, Xia S, Pan S, Qu H, Li X. 2021. Upregulation of endothelial cell-derived exosomal microRNA-125b-5p protects from sepsis-induced acute lung injury by inhibiting topoisomerase II alpha. Inflamm Res. 70(2):205–216. doi: 10.1007/s00011-020-01415-0. 33386874 PMC7776283

[CIT0018] Karki P, Birukov KG. 2019. Rho and reactive oxygen species at crossroads of endothelial permeability and Inflammation. Antioxid Redox Signal. 31(13):1009–1022. doi: 10.1089/ars.2019.7798. 31126187 PMC6765062

[CIT0019] Keiser MJ, Roth BL, Armbruster BN, Ernsberger P, Irwin JJ, Shoichet BK. 2007. Relating protein pharmacology by ligand chemistry. Nat Biotechnol. 25(2):197–206. doi: 10.1038/nbt1284. 17287757

[CIT0020] Lan KC, Chao SC, Wu HY, Chiang CL, Wang CC, Liu SH, Weng TI. 2017. Salidroside ameliorates sepsis-induced acute lung injury and mortality via downregulating NF-kappaB and HMGB1 pathways through the upregulation of SIRT1. Sci Rep. 7(1):12026. doi: 10.1038/s41598-017-12285-8. 28931916 PMC5607272

[CIT0021] Lee SY, Li MH, Shi LS, Chu H, Ho CW, Chang TC. 2013. *Rhodiola crenulata* extract alleviates hypoxic pulmonary edema in rats. Evid Based Complement Alternat Med. 2013:718739. doi: 10.1155/2013/718739. 23710233 PMC3655596

[CIT0022] Liu C, Cai B, Li D, Yao Y. 2021. Wolf-Hirschhorn syndrome candidate 1 facilitates alveolar macrophage pyroptosis in sepsis-induced acute lung injury through NEK7-mediated NLRP3 inflammasome activation. Innate Immun. 27(6):437–447. doi: 10.1177/17534259211035426. 34428935 PMC8504266

[CIT0023] Liu M, Li P, Su W. 2006. Research progress on chemical constituents and pharmacological effects of *Rhodiola*. Central South Pharm. 4:463–466.

[CIT0024] Panossian A, Seo EJ, Efferth T. 2019. Effects of anti-inflammatory and adaptogenic herbal extracts on gene expression of eicosanoids signaling pathways in isolated brain cells. Phytomedicine. 60:152881. doi: 10.1016/j.phymed.2019.152881. 30987861

[CIT0025] Pei Q, Ni W, Yuan Y, Yuan J, Zhang X, Yao M. 2022. HSP70 ameliorates septic lung injury via inhibition of apoptosis by interacting with KANK2. Biomolecules. 12(3):410. doi: 10.3390/biom12030410. 35327602 PMC8946178

[CIT0026] Piñero J, Bravo À, Queralt-Rosinach N, Gutiérrez-Sacristán A, Deu-Pons J, Centeno E, García-García J, Sanz F, Furlong LI. 2017. DisGeNET: a comprehensive platform integrating information on human disease-associated genes and variants. Nucleic Acids Res. 45(D1):D833–d839. doi: 10.1093/nar/gkw943. 27924018 PMC5210640

[CIT0027] Pu WL, Zhang MY, Bai RY, Sun LK, Li WH, Yu YL, Zhang Y, Song L, Wang ZX, Peng YF, et al. 2020. Anti-inflammatory effects of *Rhodiola rosea* L.: a review. Biomed Pharmacother. 121:109552. doi: 10.1016/j.biopha.2019.109552. 31715370

[CIT0028] Qiu N, Xu X, He Y. 2020. LncRNA TUG1 alleviates sepsis-induced acute lung injury by targeting miR-34b-5p/GAB1. BMC Pulm Med. 20(1):49. doi: 10.1186/s12890-020-1084-3. 32087725 PMC7036216

[CIT0029] Ren HH, Niu Z, Guo R, Fu M, Li HR, Zhang XY, Yao L. 2021. *Rhodiola crenulata* extract decreases fatty acid oxidation and autophagy to ameliorate pulmonary arterial hypertension by targeting inhibition of acylcarnitine in rats. Chin J Nat Med. 19(2):120–133. doi: 10.1016/S1875-5364(21)60013-4. 33641783

[CIT0030] Safran M, Dalah I, Alexander J, Rosen N, Iny Stein T, Shmoish M, Nativ N, Bahir I, Doniger T, Krug H, et al. 2010. GeneCards Version 3: the human gene integrator. Database. 2010(0):baq020–baq020. doi: 10.1093/database/baq020. 20689021 PMC2938269

[CIT0031] Seeliger D, de Groot BL. 2010. Ligand docking and binding site analysis with PyMOL and Autodock/Vina. J Comput Aided Mol Des. 24(5):417–422. doi: 10.1007/s10822-010-9352-6. 20401516 PMC2881210

[CIT0032] Sevransky JE, Martin GS, Shanholtz C, Mendez-Tellez PA, Pronovost P, Brower R, Needham DM. 2009. Mortality in sepsis versus non-sepsis induced acute lung injury. Crit Care. 13(5):R150. doi: 10.1186/cc8048. 19758459 PMC2784371

[CIT0033] Shahat AA, Ullah R, Alqahtani AS, Alsaid MS, Husseiny HA, Al Meanazel OTR. 2018. Hepatoprotective effect of *Eriobotrya japonica* leaf extract and its various fractions against carbon tetra chloride induced hepatotoxicity in rats. Evid Based Complement Alternat Med. 2018:3782768–3782768. doi: 10.1155/2018/3782768. 30643530 PMC6311294

[CIT0034] Shannon P, Markiel A, Ozier O, Baliga NS, Wang JT, Ramage D, Amin N, Schwikowski B, Ideker T. 2003. Cytoscape: a software environment for integrated models of biomolecular interaction networks. Genome Res. 13(11):2498–2504. doi: 10.1101/gr.1239303. 14597658 PMC403769

[CIT0035] Song D, Zhao M, Feng L, Wang P, Li Y, Li W. 2021. Salidroside attenuates acute lung injury via inhibition of inflammatory cytokine production. Biomed Pharmacother. 142:111949. doi: 10.1016/j.biopha.2021.111949. 34325302

[CIT0036] Sterling T, Irwin JJ. 2015. ZINC 15–ligand discovery for everyone. J Chem Inf Model. 55(11):2324–2337. doi: 10.1021/acs.jcim.5b00559. 26479676 PMC4658288

[CIT0037] Tao H, Wu X, Cao J, Peng Y, Wang A, Pei J, Xiao J, Wang S, Wang Y. 2019. *Rhodiola* species: a comprehensive review of traditional use, phytochemistry, pharmacology, toxicity, and clinical study. Med Res Rev. 39(5):1779–1850. doi: 10.1002/med.21564. 30652331

[CIT0038] Trott O, Olson AJ. 2010. AutoDock Vina: improving the speed and accuracy of docking with a new scoring function, efficient optimization, and multithreading. J Comput Chem. 31(2):455–461. doi: 10.1002/jcc.21334. 19499576 PMC3041641

[CIT0039] Wang J, Xu H. 2016. Determination of gallic acid, salidroside and tyrosol in *Rhodiola*. J Sichuan Tradit Chin Med. 34:145–147.

[CIT0040] Wang M, Luo L, Yao L, Wang C, Jiang K, Liu X, Xu M, Shen N, Guo S, Sun C, et al. 2016. Salidroside improves glucose homeostasis in obese mice by repressing inflammation in white adipose tissues and improving leptin sensitivity in hypothalamus. Sci Rep. 6(1):25399. doi: 10.1038/srep25399. 27145908 PMC4857131

[CIT0041] Wei Z. 2010. The protective effect of seven compounds from the roots of *Rhodiola sachalinesis* (A. Bor) against septic shock and its mechanism. Yanbian University. [In Chinese].

[CIT0042] Wu D, Wu K, Zhu Q, Xiao W, Shan Q, Yan Z, Wu J, Deng B, Xue Y, Gong W, et al. 2018. Formononetin administration ameliorates dextran sulfate sodium-induced acute colitis by inhibiting NLRP3 inflammasome signaling pathway. Mediators Inflamm. 2018:3048532. doi: 10.1155/2018/3048532. 29507526 PMC5817291

[CIT0043] Xu KJ, Zhang SF, Li QX. 2003. [Preventive and treatment effect of composite *Rhodiolae* on acute lung injury in patients with severe pulmonary hypertension during extracorporeal circulation]. Zhongguo Zhong Xi Yi Jie He Za Zhi. 23:648–650. 14571608

[CIT0044] Yang R, Yang H, Wei J, Li W, Yue F, Song Y, He X, Hu K. 2021. Mechanisms underlying the effects of Lianhua Qingwen on sepsis-induced acute lung injury: a network pharmacology approach. Front Pharmacol. 12:717652. doi: 10.3389/fphar.2021.717652. 34721017 PMC8551812

[CIT0045] Yang W, Zhang W, Yang Y, Li D, Liu Z. 2015. General situation research progress on *Rhodiola rosea* chemical composition. Capital Med. 22:90–91.

[CIT0046] Yuan Y, Zhang L, Li Y. 2007. Active components and pharmacological action of integripetal *Rhodiola* herb. Food Drug. 9:54–57.

[CIT0047] Zhang GB, Li QY, Chen QL, Su SB. 2013. Network pharmacology: a new approach for Chinese herbal medicine research. Evid Based Complement Alternat Med. 2013:621423. doi: 10.1155/2013/621423. 23762149 PMC3671675

[CIT0048] Zhang H, Dong W, Li S, Zhang Y, Lv Z, Yang L, Jiang L, Wu T, Wang Y. 2021. Salidroside protects against ventilation-induced lung injury by inhibiting the expression of matrix metalloproteinase-9. Pharm Biol. 59:760–768. 34517742 10.1080/13880209.2021.1967409 PMC8439245

[CIT0049] Zhang J, Jin S, Geng B, Li H, Zeng C, Xu L, Gu D, Li Y, Gu Z. 2016. Determination of six flavonoids in *Rhodiolae* Crenulatae Radix et Rhizoma by HPLC-DAD. Chin Tradit Herbal Drugs. 47:4253–4256.

[CIT0050] Zhang K, Si XP, Huang J, Han J, Liang X, Xu XB, Wang YT, Li GY, Wang HY, Wang JH. 2016. Preventive effects of *Rhodiola rosea* L. on bleomycin-induced pulmonary fibrosis in rats. Int J Mol Sci. 17(6):879. doi: 10.3390/ijms17060879. 27271612 PMC4926413

[CIT0051] Zhang X, Kuang G, Wan J, Jiang R, Ma L, Gong X, Liu X. 2020. Salidroside protects mice against CCl_4_-induced acute liver injury via down-regulating CYP2E1 expression and inhibiting NLRP3 inflammasome activation. Int Immunopharmacol. 85:106662. doi: 10.1016/j.intimp.2020.106662. 32544869

[CIT0052] Zhang Y, Zang B, Li X, Zhao W. 2017. Effect of sofren injection on NF-κB expression in acute lung injury mice with sepsis. J Clin Pulm Med. 22:2147–2150.

[CIT0053] Zhou B, Weng G, Huang Z, Liu T, Dai F. 2018. Arctiin prevents LPS-induced acute lung injury via inhibition of PI3K/AKT signaling pathway in mice. Inflammation. 41(6):2129–2135. doi: 10.1007/s10753-018-0856-x. 30116933

