# Supplementary material for: Elucidating the role of Rhodiola rosea L. in sepsis-induced acute lung injury via network pharmacology: emphasis on inflammatory response, oxidative stress, and the PI3K-AKT pathway
Source: Pharm Biol. 2024 Mar 6;62(1):272–84. doi: 10.1080/13880209.2024.2319117 (PMC10919309; doi:10.1080/13880209.2024.2319117)
Supplement: Supplemental Material [file IPHB_A_2319117_SM3251.zip › table S1.docx]

Table S1: 49 key targets of *Rhodiola rosea* L. against sepsis-induced acute lung injury

| **ID** | **Name** | **ID** | **Name** | **ID** | **Name** | **ID** | **Name** |
| --- | --- | --- | --- | --- | --- | --- | --- |
| 3725 | JUN | 2099 | ESR1 | 4353 | MPO | 5054 | SERPINE1 |
| 5243 | ABCB1 | 2100 | ESR2 | 4714 | NDUFB8 | 6513 | SLC2A1 |
| 4363 | ABCC1 | 2152 | F3 | 4780 | NFE2L2 | 4088 | SMAD3 |
| 100 | ADA | 2247 | FGF2 | 1728 | NQO1 | 7015 | TERT |
| 231 | AKR1B1 | 2322 | FLT3 | 4893 | NRAS | 7097 | TLR2 |
| 217 | ALDH2 | 2671 | GFER | 5320 | PLA2G2A | 54106 | TLR9 |
| 240 | ALOX5 | 2936 | GSR | 5321 | PLA2G4A | 7132 | TNFRSF1A |
| 595 | CCND1 | 2944 | GSTM1 | 5468 | PPARG | 7276 | TTR |
| 1376 | CPT2 | 3265 | HRAS | 5914 | RARA | 7422 | VEGFA |
| 2833 | CXCR3 | 3558 | IL2 | 5915 | RARB | 7498 | XDH |
| 2033 | EP300 | 3569 | IL6 | 6401 | SELE |  |  |
| 2052 | EPHX1 | 4151 | MB | 6402 | SELL |  |  |
| 2053 | EPHX2 | 4282 | MIF | 6403 | SELP |  |  |
