# Supplementary material for: Elucidating the role of Rhodiola rosea L. in sepsis-induced acute lung injury via network pharmacology: emphasis on inflammatory response, oxidative stress, and the PI3K-AKT pathway
Source: Pharm Biol. 2024 Mar 6;62(1):272–84. doi: 10.1080/13880209.2024.2319117 (PMC10919309; doi:10.1080/13880209.2024.2319117)
Supplement: Supplemental Material [file IPHB_A_2319117_SM3251.zip › table S2.docx]

Table S2: 22 main active components of *Rhodiola rosea* L.

| **name** | **GI absorption** | **Lipinski** | **Ghose** | **Veber** | **Egan** | **Muegge** | **Bioavailability** |
| --- | --- | --- | --- | --- | --- | --- | --- |
| arbutin | High | 1 | 0 | 1 | 1 | 1 | 0.55 |
| linolenate | High | 1 | 0 | 0 | 1 | 0 | 0.85 |
| n-Hexanol | High | 1 | 0 | 1 | 1 | 0 | 0.55 |
| scopoletin | High | 1 | 1 | 1 | 1 | 0 | 0.55 |
| 1-Decanol | High | 1 | 0 | 1 | 1 | 0 | 0.55 |
| 1-Octanol | High | 1 | 0 | 1 | 1 | 0 | 0.55 |
| 1-Octen-3-ol | High | 1 | 0 | 1 | 1 | 0 | 0.55 |
| 2-(4-Hydroxyphenyl)ethanol | Hig­­­­h | 1 | 0 | 1 | 1 | 0 | 0.55 |
| berchemol | High | 1 | 1 | 1 | 1 | 1 | 0.55 |
| Butyric acid | High | 1 | 0 | 1 | 1 | 0 | 0.85 |
| coumarin | High | 1 | 0 | 1 | 1 | 0 | 0.55 |
| Ellagic acid | High | 1 | 1 | 0 | 0 | 1 | 0.55 |
| Gallic acid | High | 1 | 0 | 1 | 1 | 0 | 0.56 |
| Herbacetin | High | 1 | 1 | 1 | 1 | 1 | 0.55 |
| kaempferol | High | 1 | 0 | 1 | 1 | 1 | 0.55 |
| Myrtenol | High | 1 | 0 | 1 | 1 | 1 | 0.55 |
| palmitate | High | 1 | 1 | 0 | 1 | 0 | 0.85 |
| Quercetin | High | 1 | 1 | 1 | 1 | 1 | 0.55 |
| Salidroside | High | 1 | 0 | 1 | 1 | 1 | 0.55 |
| scaphopetalone | High | 1 | 1 | 1 | 1 | 1 | 0.55 |
| Syringic acid | High | 1 | 1 | 1 | 1 | 0 | 0.56 |
| umbelliferone | High | 1 | 0 | 1 | 1 | 0 | 0.55 |

In this table, 1 means that the compound meets the requirements of the pharmacopoeia rules for the class, whereas 0 means that it does not meet the requirements of the pharmacopoeia rules for the class
