# Supplementary figures and images for: Elucidating the role of Rhodiola rosea L. in sepsis-induced acute lung injury via network pharmacology: emphasis on inflammatory response, oxidative stress, and the PI3K-AKT pathway
Source: Pharm Biol. 2024 Mar 6;62(1):272–84. doi: 10.1080/13880209.2024.2319117 (PMC10919309; doi:10.1080/13880209.2024.2319117)

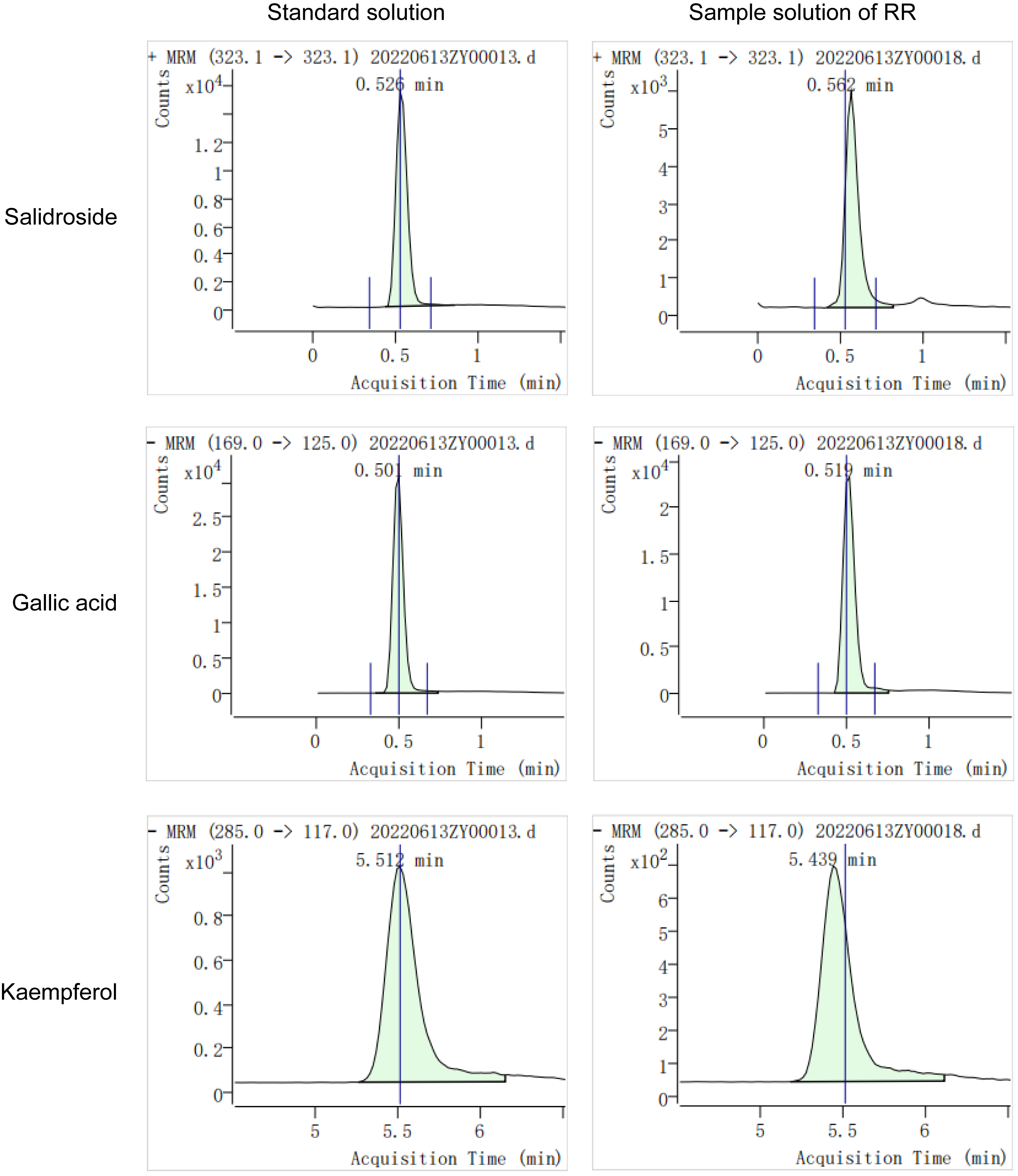

Supplement: Supplemental Material [file IPHB_A_2319117_SM3251.zip › Figure S1.tif]

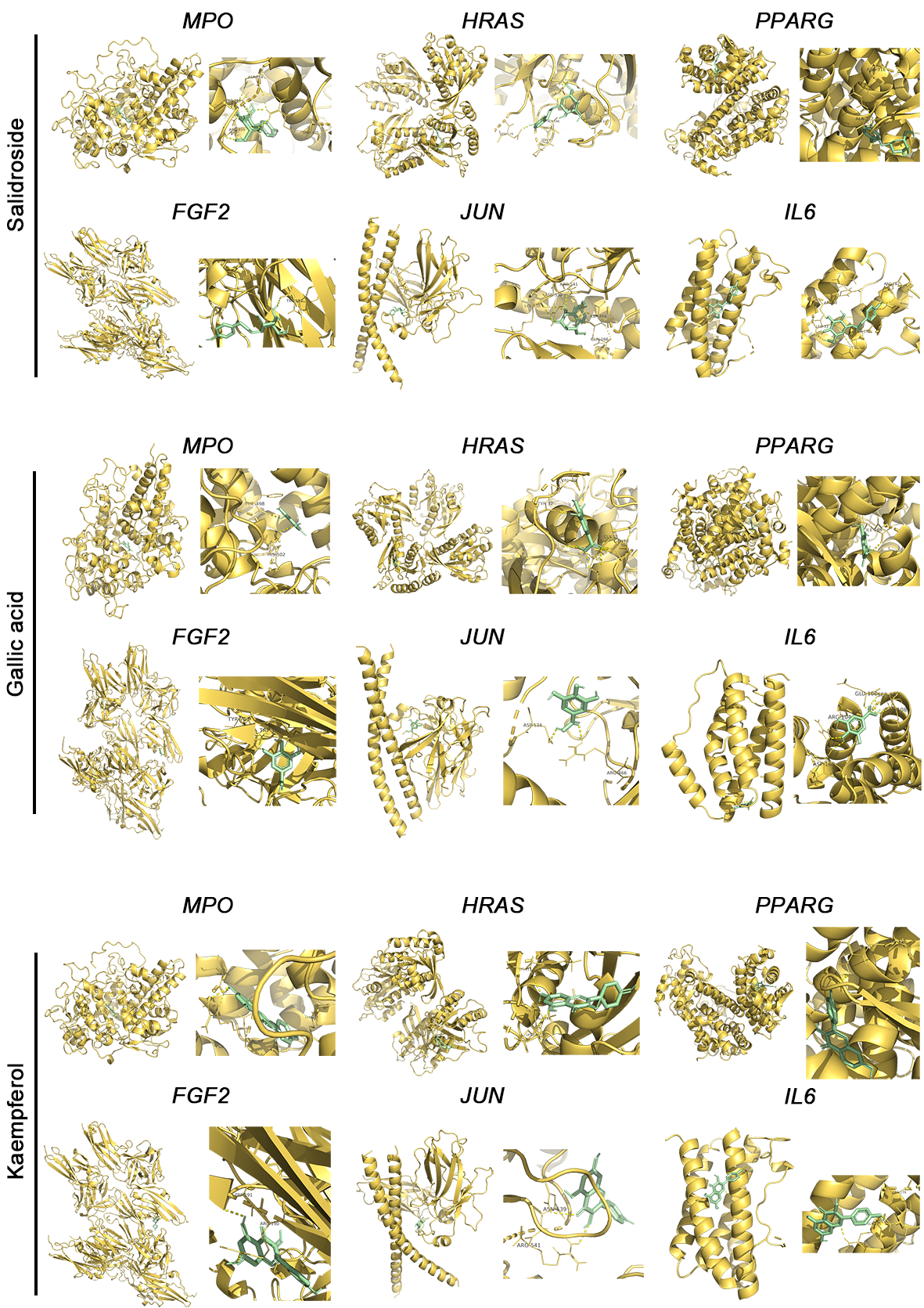

Supplement: Supplemental Material [file IPHB_A_2319117_SM3251.zip › Figure S2.tif]
